# Tackling the various classes of nano-therapeutics employed in topical therapy of psoriasis

**DOI:** 10.1080/10717544.2020.1754527

**Published:** 2020-05-12

**Authors:** Salma A. Fereig, Ghada M. El-Zaafarany, Mona G. Arafa, Mona M. A. Abdel-Mottaleb

**Affiliations:** a Faculty of Pharmacy, Department of Pharmaceutics and Pharmaceutical Technology, The British University in Egypt (BUE), El Sherouk City, Egypt;; b Faculty of Pharmacy, Department of pharmaceutics and Industrial Pharmacy, Ain Shams University, Cairo, Egypt;; c Chemotherapeutic Unit, Mansoura University Hospitals, Mansoura, Egypt

**Keywords:** Hybrid, lipid, metal, polymers, psoriasis, topical

## Abstract

Psoriasis is a dermatological chronic skin condition with underlying autoimmune etiology. It deeply affects patients’ quality of life. Therefore, it was an interesting target for researchers throughout the past years. Conventionally, the treatment options include anti-inflammatory agents, immune suppressants, biologic treatment, and phototherapy. Nanotechnology offers promising characteristics that allow for tailoring a drug carrier to achieve dermal targeting, improved efficacy and minimize undesirable effects. Being the safest route, the first line of treatment and a targeted approach, we solely discussed the use of the topical route, combined with advanced drug delivery systems for the management of psoriasis in this article. Advanced systems include polymeric, metallic, lipidic and hybrid nanocarriers incorporating different active agents. All formerly mentioned types of drug delivery systems were investigated through the past decades for the purpose of topical application on psoriatic plaques. Scientists’ efforts are promising to reach an optimized formula with a convenient dosage form to improve efficacy, safety, and compliance for the treatment of psoriasis. Accordingly, it will offer a better quality of life for patients.

## 1. Introduction

### 1.1. Psoriasis

Psoriasis is a widespread disease categorized as chronic autoimmune inflammation of the skin (Sala et al., 2016). According to the WHO, it distresses 1.5–5% of the population in developing countries and 0.9–11.4% worldwide (Michalek et al., 2016). It is usually manifested by erythematous or scaly lesions that may be localized at any part of the body, often appearing at the scalp and joints, the spread of these lesions varies according to the severity of the condition (Harden et al., 2015). The exact causes of the disease are still unknown but it may be attributed to various factors including family history, tobacco smoking, alcohol consumption, and stress.

**Figure 1.**
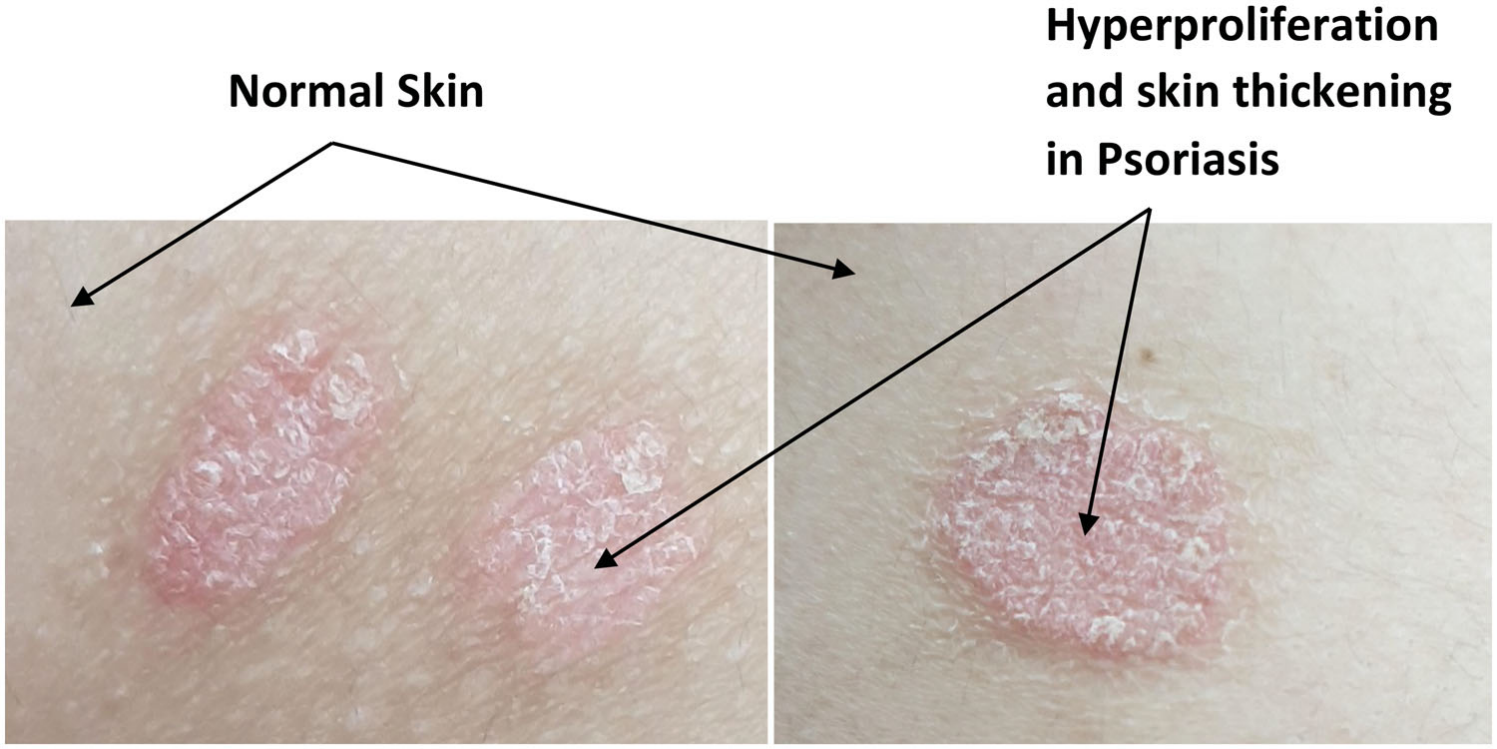
Psoriatic plaques covered with silvery scales compared to normal skin parts.

Psoriasis’ patients suffer from psychological burdens due to visible disfiguration of erythematous skin lesions covered by silvery scales (Figure 1). These manifestations may lead to depression and suicidal attempts owing to the distorted quality of life (Wan et al., 2017; González-Parra & Daudén, 2019). The major role of immune cells in the pathogenesis of psoriasis was confirmed on using immune-suppressants as a treatment approach which showed significant improvement of the disease’s manifestations.

**Figure 2.**
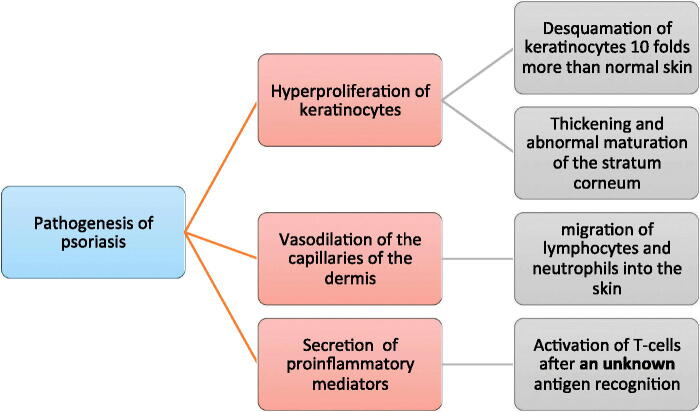
A summary of the underlying pathogenesis of psoriatic skin lesions.

The underlying pathogenic causes of psoriasis are summarized in Figure 2. It includes three main lines; first, the hyperproliferation of keratinocytes as the psoriatic skin cell lifetime is more than six times shorter than that of normal skin cells, this is thought to be due to hyperactivity of growth factors (Das et al., 2009). Second, angiogenesis, the exact mechanism of this process is still vague to scientists, but it is known to cause the widening of intercellular spaces and blood capillaries dilation leading to the production of pro-inflammatory cytokines, which is the third line of pathogenesis. These pro-inflammatory mediators include interleukins, endothelin, IFN-ϒ, TNF-α and VEGF (vascular endothelial growth factor). They are thought to activate T-cells, probably by an unknown antigen which leads to an immune response that is memorized then by memory cells, leading to a cross-reaction on exposure to an autoantigen (Rahman et al., 2012).

Types of psoriasis include plaque, presented with red plaques with well-defined edges and a pearl-like layer, pustular, where the lesions include boils, Guttate, usually a complication of bacterial infection and the lesions appear commonly as small drops, and inverse, a type in which the affected area is only skin folds like underarm and groin area (Sarac et al., 2016). Eighty percent of psoriasis’ patients are presented with plaque psoriasis (Sticherling, 2016). Psoriasis is also categorized according to the severity of the case into mild, moderate or severe.

### 1.2. Conventional management of psoriasis

Management of psoriasis comprises four main approaches: topical treatment, phototherapy, systemic treatment and biological systemic treatment that targets specific receptors of psoriasis’ inflammatory pathway. Topical treatment is generally considered as a first-line treatment in mild –to moderate localized psoriasis. However, it is used as well in case of moderate to severe psoriasis but not as monotherapy; usually combined with phototherapy or systemic therapy. Topical agents include; corticosteroids (anti-inflammatory agents that inhibit the transcription of pro-inflammatory mediators like IL-2), dithranol (a suppressor for skin hyperproliferation and granulocyte function), vitamin D analogs (as immunomodulators), tacrolimus (a powerful immunosuppressive agent) and retinoids which interfere in the cytokine-regulated inflammatory pathway. The main drawback of this category, topical treatment, is the lack of sustained elimination of lesions which may be disappointing for patients (Kragballe, 1989; Kemény et al., 1990; Saurat, 1999; Kim, 2010; Higgins, 2017).

Phototherapy, the use of ultraviolet radiation with a wavelength that ranges from 254 to 313 nm, is recommended for moderate to severe psoriasis and for patients who have shown resistance to topical treatment. The UV radiation within this range has shown erythema and damage to normal skin despite it has shown therapeutic effects on psoriatic skin which may be attributed to the pathological skin changes that take place, including the thickening and keratinization of the epidermal skin layer. UVB is most commonly used if phototherapy is the approach of choice because of its safety, efficacy, and cost-effectiveness. PUVA (Psoralen + UVA) is also used but it is usually not preferable because of the potential associated risks including; photoaging, squamous cell carcinoma or even malignant melanoma. However, it is very effective and minimizes recurrence for a relatively long period of time (Parrish & Jaenicke, 1981; Menter et al., 2008).

The most commonly prescribed agents by systemic route include; methotrexate, cyclosporine, acitretin and fumaric acid ester, an immunomodulatory with antioxidative properties. Methotrexate, a folic acid synthesis inhibitor that has immunosuppressive and anti-inflammatory properties, is considered as a first-line in this category due to its relatively high safety profile compared to other systemic agents and it shows high efficacy in most cases. However, it may cause hepatotoxicity, hence close monitoring of liver functions is recommended in case of prolonged treatment and is totally contraindicated in case of any existing liver illness. It is also contraindicated in the case of pregnancy, thrombocytopenia, and leukemia. Cyclosporine, being a potent immunosuppressant, makes it an effective agent for the management of psoriasis but the risk of glomerulosclerosis, lymphoma, renal impairment, and hypertension have limited its use to mainly short-term courses. Acitretin, a retinoid, is also an effective agent that is used in minor doses because of its teratogenic effect and the potential risk of dyslipidemia. It is often prescribed in combination with phototherapy (Silverman et al., 1987; Buchman, 2001; Czarnecka-Operacz & Sadowska-Przytocka, 2014; Balak, 2015).

Biological systemic treatment targets specific receptors of the psoriasis’ inflammatory pathway particularly by two pathways; inhibition of T-cell (e.g. Alefacept, Efalizumab) and inhibition of Tumor necrosis factor (e.g. Infliximab, Etanercept, Adalimumab). The global guidelines recommend specific laboratory tests before initiating this therapy and follow-up tests as well including; complete blood cell count, liver functions and screening for hepatitis, tuberculosis, human immune deficiency virus as well as pregnancy. The risk of infections should be minimized and vaccination is prohibited. Thus, this category is considered the last option as it targets the immune system and may cause the future impaired response to any infection (Salvadori & Bertoni, 2003).

Natural agents have shown promising outcomes in the treatment of psoriasis as well, especially when combined with antipsoriatic chemical agents (Murphy et al., 2019; Bakshi et al., 2020). Examples of natural substances that were reported to have beneficial antipsoriatic properties include curcumin, capsaicin, fish oil, and green tea, berries’ and aloe Vera extracts (Agarwal et al., 2001; Crisan et al., 2018; Murphy et al., 2019; Chen et al., 2020). Fish oil components particularly have shown beneficial therapeutic effects against psoriatic plaques when used solely or in combination with other chemical agents. It is claimed to be a very good candidate for Nanoparticulate delivery system to achieve the desired response (Rahman et al., 2013).

The choice of treatment is dependent on co-morbidities and the severity of the disease. As topical treatment is considered the first-line, most researchers have focused on this route of administration to improve its efficacy and safety. The aforementioned drugs and drug combinations work by different pathways including targeting eicosanoids, chemokines, cAMP, T-cells, gene transcription or ROS (reactive oxygen species) (Rahman et al., 2015).

The systemic route is not preferable for the management of psoriasis as the active ingredients used for this purpose may have serious side effects such as cytotoxicity and immunosuppression (Buchman, 2001; Menter et al., 2008; Czarnecka-Operacz & Sadowska-Przytocka, 2014; Balak, 2015). Hence, the topical route is considered to be very advantageous in the case of psoriasis compared to the systemic route. It is the most convenient for the patient, improves compliance and minimizes side effects. These features are crucial in the management of psoriasis as it is a chronic disease which means that these formulas are being used for very long time periods if not for the patients’ whole lifetime (Harden et al., 2015; Higgins, 2017; Musa et al., 2017). Besides, the topical route is stated to be the first line in the management of psoriasis (Rahman et al., 2012; Golbari et al., 2018. Accordingly, we have chosen to restrict this article to the topical approaches in particular.

### 1.3. Nanotechnology

**Figure 3.**
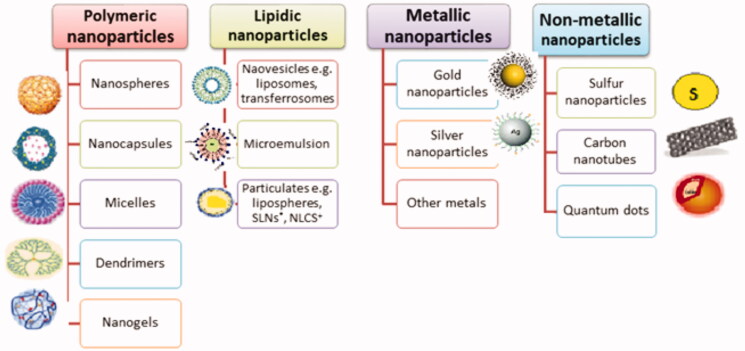
Various types of nanocarriers used for topical drug delivery.

Upon minimizing the particle size to the nanometer range, particles tend to show different characteristics from those of the original larger ones (Abdel-Mottaleb et al., 2014). Over the past few decades, the science of nanotechnology has gained great interest from researchers in various fields including drug delivery (Goyal et al., 2016). The use of nanotechnology offers many advantages over conventional drug delivery systems such as modifying solubility of hydrophobic materials, achieving controlled or sustained release, promoting drugs’ stability and targeted therapy to the site of action which increases efficacy and minimizes side effects. Several nanocarriers were investigated for topical drug delivery including polymeric, lipidic and metallic ones (Rahman et al., 2018; Figure 3). In this article, we will be discussing the different advanced drug delivery systems that were previously investigated as topical dosage forms for local treatment of psoriatic skin plaques in the past few years.

Fick’s described topical drug delivery in his second law, stating that the transcutaneous flux of a drug is inversely proportional to the thickness of the stratum corneum and directly proportional to the drug concentration (Goyal et al., 2016). Psoriatic skin functions and how the pathological condition of the skin minimized or exceeded the barrier function of the stratum corneum and epidermis was a point of a question for researchers for many years. Early in 1996, Ghadially et al. had studied the disruption of the barrier function of the skin in different types of psoriasis Using TEWL (Transepidermal water loss) (Ghadially et al., 1996). They assume that the barrier function of the skin was defective, based on previous research tackling this point in 1945, 1967 and 1973. However, the homeostasis functions of the body compensate for this phenomenon by the excessive skin cells’ replication (forming scales) in the case of chronic plaque psoriasis. Thus, they describe the aforementioned process to be a possible underlying cause of chronic psoriasis. Takahashi and his fellows have proven experimentally that psoriatic skin contains less skin water content, natural moisturizing factor, and free fatty acids compared to normal skin (Takahashi et al., 2014). In contrast, sebum content was not affected by psoriasis. Sano, states that psoriatic skin has diminished ceramide levels when examined by immunostaining (Sano, 2015).

Hyperproliferation of keratinocytes leads to abnormal thickening of the stratum corneum as shown in Figure 1, thus topical drug delivery to psoriatic skin presents a challenge to formulators, particularly, due to hindered skin penetration (Pradhan et al. 2018; Singhvi et al., 2020). Lin Sun, has tested the penetration of nanoparticles in imiquimod-induced psoriasis in mouse models. Imiquimod treated skin has shown improved skin permeation and retention than normal mice skin (Sun et al., 2018). Thus, the literature confirms that skin suffers major structural and functional changes in the case of psoriasis which directly affects drug delivery and retention.

In accordance, nanoparticles do offer advantageous properties compared to conventional treatment. They not only provide the facility of tailoring the carrier to the desired need either by controlling size, combining hydrophobic drugs in hydrophilic carriers or adding a targeting moiety but they also deliver higher concentrations of the drug-using enhanced permeation retention (EPR) effect and controlled drug release. Poor efficacy of conventional topical treatment, due to poor permeation, has always left physicians with no choice but to use systemic approaches which leads to the aforementioned side effects and poor patients’ compliance (Rapalli et al., 2018). The use of nanotechnology approaches has the potential to overcome these problems and penetrate the horny psoriatic layer and be retained in the epidermis by different mechanisms as will be discussed below.

## 2. Advanced nanotechnology-based approaches for topical treatment of psoriatic plaques

### 2.1. Polymeric nanoparticles

Polymeric nanoparticles are colloidal structures made of macromolecules with a particle size ranging from 10–1000 nm (Jawahar & Meyyanathan, 2012; Venditti, 2017). They are usually preferred by formulators as nanocarriers for drug delivery and targeting due to their flexibility in structure by chemical modification, suitability for different types of drugs, long circulation time in the body, biocompatibility, biodegradability, nonimmunogenicity, and availability in a variety of formulation methods for preparation (Bhatia, 2016).

There are diverse types of polymeric nanoparticles; nanospheres, nanocapsules, dendrimers, polymeric micelles and nanogels (Soni et al., 2016). Nanospheres are matrix-based structures in which the drug is either dispersed within the polymer matrix or adsorbed on the surface of the sphere. Nanocapsules are core/shell-based structures where the core is composed of a liquid suspension containing the drug and the polymer constitutes the shell. The drug may also be adsorbed to the surface of the nanocapsule (Jawahar & Meyyanathan, 2012). On the other side, dendrimers are usually synthesized using polymers with a highly branched 3D structure as polyamidoamine (PAMAM) with the active constituent covalently or electrostatically bonded to the core or surface (Venditti, 2017). As for polymeric micelles, they are synthesized from amphiphilic block copolymers with structures very similar to surfactant micelles with higher stability in physiological solutions. Nanogels are hydrophilic crosslinked polymeric networks that have a high capacity to incorporate molecules, they are responsive to external stimuli which makes them easily tailored by the proper choice of polymer and crosslinker (Soni et al., 2016). All types of polymeric carriers can incorporate both hydrophilic and hydrophobic drugs based on the choice of polymer and method of preparation. There are many different types of natural and synthetic polymers with diverse characteristics. These polymers and their documented applications are summarized in (Table 1).

**Table 1.** A summary of polymers that are commonly used for nano drug delivery targets.

Polymer	Characteristics	Application
PLGA	One of the first and most commonly used polymers in drug delivery First used in the mid 1980s FDA approved Biocompatible Biodegradable	Used for many drug delivery applications including: vaccination, cancer (Danhier et al., 2012)
PEG	A hydrophilic polymer Generally safe First used in the late 1960s	Used as a coating material for nanoparticles to achieve more efficient drug delivery by extending the drug’s circulation time in the bloodstream; to avoid elimination by RES "stealthing" (Suk et al., 2016)
Poloxamer/ PEO-PPO-PEO	A block co-polymer Amphiphilic nature Its molecular weight can be customized according to application Can arrange in solution to form polymeric micelles	Widely used for drug delivery and medical imaging Application and characteristics differ according to molecular weight ((Moghimi and Hunter, 2000)
Polyplexes:	A combination of a cationic polymer and nucleic acid therapeutics PEI: A cationic polymer, accused of high toxicity Protamine: A natural polypeptide; composed of cationic arginine units Safer than PEI but less efficient FDA approved	Used as non-viral vectors for gene delivery (Tros de Ilarduya et al., 2010)
Chitosan	A natural hydrophilic cationic polysacccahride FDA approved Biocompatible Biodegradable Derived from crustacean shells	Used extensively for drug delivery Chitosan nanoparticles are positively charged and mucoadhesive and achieve sustained drug release ((Mohammed et al., 2017)
SF	Natural biopolymer Biodegradable Biocompatible Minor immunogenicity	Nanoparticles can be synthesized from this polymer by various methods for treatment of cancer and other diseases (Gianak et al., 2018)
Albumin	Natural biopolymer Hydrophilic Mostly suitable for hydrophilic drugs	Used extensively in drug delivery and medical imaging; there are many marketed formulations for treatment of cancer, diabetes, multiple sclerosis and other conditions (An and Zhang, 2017)
Gelatin	Natural biopolymer Hydrophilic Mostly suitable for hydrophilic drugs	Used for drug and gene delivery for conditions such as; cancer, tuberculosis and human immunodeficiency viral infection (Yasmin et al., 2016)
Dextran	Natural biopolymer Hydrophilic Mostly suitable for hydrophilic drugs	Often used with functionalization for specific targeting, for example, the CNS or liver cells (Foerster et al., 2016; Ibegbu, 2015; Liu et al., 2018)
PLA-PCL-PGA	Forms polymeric micelles Biodegradable Synthetic PLA is hydrophilic PCL is Hydrophobic PGA is Hydrophilic	PGA, PLA and PCL and their copolymers are the most commonly used materials for nanoparticles synthesis for the purpose of drug delivery (Hans and Lowman, 2002)
PAMAM/PPI	Used as a dendritic platform PPI is the first-discovered dendrimer	Used to incorporate hydrophobic drugs in particular, as they improve their solubility and control their release (Huang and Wu, 2018)
PHPMA	A non-biodegradable synthetic polymer	Synthesis of new drug delivery systems with tailored characteristics (Huang and Wu, 2018)
PACA	Synthesized by anionic polymerization Biodegradable Biocompatible FDA approved	Used alone or with a copolymer for the purpose of drug delivery for cancer or other diseases. Widely used for delivery of different therapeutic agents in different forms of nanocarriers including: nanocapsules, nanospheres, long circulating nanoparticles and nanoparticles conjugated with targeting moieties (Yordanov, 2012)

PLGA: polylactic co-glycolic acid; PEG: polyethylene glycol; PEO-PPO-PEO: polyethylene oxide-polypropylene oxide-polyethylene oxide; PEI: polyethyleneimine; SF: silk fibroin; PLA-PCL-PGA: polylactic acid-polycaprolactone-polyglycolic acid; PAMAM: polyamidoamine; PPI: polypropyleneimine; PHPMA: polyhydroxylpropylmethacrylamide; PACA: polyalkylcyanoacrylates.

Polymeric nanoparticles, either nanospheres or nanocapsules, show significant drug accumulation in the stratum corneum layer of the skin compared to emulsion type nanosystems, which tend to accumulate in the dermis layer (Alves et al., 2007; Guterres et al., 2007). Moreover, polymerics do deposit in the skin more than lipidic nanosystems, suggesting that the former is suitable for topical treatment while the latter is more appropriate for transdermal (Abdel-Mottaleb et al., 2011; Abdel-Mottaleb & Lamprecht, 2016). Charged polymeric nanoparticles, in particular, have higher retention time on inflamed skin leading to a superior therapeutic outcome (Abdel-Mottaleb et al., 2012b). In addition, their selective accumulation in inflamed skin layers in particular (Abdel-Mottaleb et al., 2012a). Thus, it is suggested that polymerics are the most suitable drug delivery systems for local psoriasis treatment, which involves skin inflammation in the outermost layer, stratum corneum. Studies that investigated using polymeric nanoparticles for topical treatment of psoriatic plaques are summarized in Table 2.

**Table 2.** A summary of recent approaches carried out to formulate drug delivery systems for topical management of psoriasis using polymeric nanoparticles.

Drug	Drug delivery system	Method of preparation	Findings
Tretinoin	Lipid-core PCL nanocapsules	Interfacial deposition	Decreased skin permeation and photodegradation (Ourique, 2011)
	PCL lipid-core nanocapsules	Interfacial deposition	Enhanced antiproliferative activity (a significant decrease in the mitotic index) (FACHINETTO et al., 2008)
Tacrolimus	Chitosan-Nicotinamide nanoparticles Hyaluraunic acid-cholesterol-nicotinamide nanocarriers	Ionic gelation Ultrasonic cell disruption of a nicotinamide solution of tacrolimus and hyalauronic-acid cholesterol conjugate	Better drug solubility, EE and stabilityEnhanced permeability (Yu et al., 2017) Improved solubility and facilitated drug loading, Enhanced skin permeation (Gabriel et al., 2016; Wan et al., 2017)
	mPEG-hexPLA nanoparticles	Emulsion solvent diffusion method	Higher drug loading, Better accumulation in the SC (Gabriel et al., 2016)
Curcumin	PLGA nanoparticles	Anti-solvent and flash precipitation	Accumulation in the SC and sustained release (Sun et al., 2017)
	RRR-α-tocopheryl succinate-grafted-ε-polylysine conjugate	Dialysis-homogenization	Sustained release and enhanced efficacy by an occlusive effect through skin hydration (Mao et al., 2017)
Nile red as a model for lipophilic compounds	Core multishell dendritic carriers	Modified film uptake	A promising biocompatible carrier for antipsoriasis agents (Pischon et al., 2017)
Clobeatsol propionate	Lecithin-chitosan nanoparticles	Direct injection of soybean lecithin ethanolic solution into chitosan solution	Better skin accumulation, Low transdermal delivery (Şenyiğit et al., 2010)
	PLGA microparticulates	Emulsion solvent evaporation	Improved *in vitro* antipsoriatic activity (Badıllı et al., 2011)
	PCL lipid-core nanocapsules	Interfacial deposition	Improved anti-inflammatory efficacy (Fontana et al., 2011)
Cyclosporin A	Micellar nanocarriers	Solvent evaporation method	Better SC deposition and solubility (Lapteva et al., 2014)
	PLGA nanoparticles	Modified emulsion-diffusion-evaporation Method	Better percutaneous delivery Minimized transdermal side effects (Jain et al., 2011)
Methotrexate	PolyNIPAM-co-BA Nanogel	Surfactant-free emulsion polymerization	PH-responsive formula Better skin accumulation with a minimum lag time Minimized transdermal side effects (Singka et al., 2010)
Hydrocortisone	PCL nanoparticles	Modified solvent displacement method	Good polymer biocompatibility (Rosado et al., 2013)

EE: entrapment efficiency; SC: stratum corneum; PolyNIPAM-co-BA: poly N-isopropylacrylamide co-butylaccrylate.

The therapeutic efficiency and stability of various drugs have been significantly improved by the use of different types of polymeric nanocarriers. Ourique (2011) prepared lipid-core PCL nanocapsules incorporating tretinoin, an active metabolite of vitamin A, in a carbopol hydrogel and compared it to a carbopol hydrogel containing non-encapsulated tretinoin with the aim of increasing the retention time on the outer skin layer. The encapsulated formula also showed better photostability on exposure to UVA and UVC radiations compared to the free drug solution indicating an improved photostability which is considered a major limitation of the drug. In addition, Fachinetto et al. (2008) also formulated tretinoin in the same drug delivery system and tested its antiproliferative effects on onion-root tip cells in comparison to the free drug, the results showed higher antiproliferative activity of the encapsulated drug demonstrated by a decrease in the mitotic index.

Researchers explored using Clobetasol propionate (CP) in a novel drug delivery system to treat dermatological disorders like psoriasis and atopic dermatitis. CP is an extremely potent corticosteroid that causes rapid healing of psoriatic lesions though, the high incidence of side effects has limited its use (Badıllı et al., 2011; Fontana et al., 2011). Ulya Badilli et al. (Badıllı et al., 2011) loaded the potent drug on PLGA microspheres emulgel and compared it to the commercially marketed product containing the free drug to find out that encapsulation of the drug has enhanced the *in vitro* release results. In another study, Şenyiğit et al. (Şenyiğit et al., 2010) used lecithin-chitosan nanoparticles as a carrier for CP in a chitosan gel to improve skin accumulation over the commercial product and lower its systemic absorption, hence minimizing systemic side effects. Furthermore, Fontana et al. (Fontana et al., 2011), fabricated lipid-core PCL nanoparticle hydrogel system and proved a significant decrease in NTPDase, nucleoside triphosphate diphosphohydrolase, activity *in vivo* (an enzyme that was reported to have a crucial role in cytokine expression, inflammatory response, cell proliferation, and apoptosis). This study aimed at the treatment of atopic dermatitis, however, the approach may also be useful in psoriasis due to the common pathological inflammatory response.

Tacrolimus, a potent immunosuppressive macrolide isolated from Streptomyces tsukubaensis, is usually prescribed both topically and systemically to psoriasis patients. Due to its hydrophobic nature, the drug is usually incorporated in a greasy base which leads to poor patient compliance due to discomfort, as well as, poor drug deposition in hyperkeratotic psoriatic plaques (Gabriel et al., 2016). Hence, the incorporation of Tacrolimus into various forms of nanosystems such as microemulsion, ethosomes, lipid nanoparticles, modified nanolipid carrier, and polymerics has been thoroughly investigated. Gabriel et al. (2016) formulated tacrolimus in a polymeric nanocarrier system using methoxy poly(ethylene glycol)-hexyl substituted poly (lactic acid) (mPEGhexPLA) as the polymer carrier. The polymer had an amphiphilic nature that enabled higher loading capacity of the hydrophobic drug, in addition to its ability to self-assemble in an aqueous environment of the hydrogel system which in turn improved patients’ compliance. On comparing the histopathology of skin layers treated with Tacrolimus nanoparticles as a hydrogel with those treated with clobetasol propionate, and the tacrolimus ointment, the nanoparticles hydrogel system showed an increase in drug deposition in the stratum corneum, viable epidermis and upper dermis with no systemic absorption. This indicated that the nanoparticle formulation improved both the efficacy and safety profiles of the potent drug. Wan et al. (Wan et al., 2017; Yu et al., 2017) also investigated modifying the aqueous solubility of Tacrolimus via complexation with hydrotropic nicotinamide, then formulated it in chitosan and hyaluraunic acid-cholesterol nanoparticles in order to improve its percutaneous delivery for treatment of psoriasis and atopic dermatitis. The hyaluronic acid-cholesterol-nicotinamide formula showed a significant improvement in psoriatic symptoms such as erythema, scales, and skin thickness.

Cyclosporin A, a potent immunosuppressive macrolide that targets skin T cells is the first line of treatment of severe psoriasis (Lapteva et al., 2014; Pardasani et al., 2000). It was formulated in the form of PLGA nanoparticles by Jain et al. (Jain et al., 2011) in order to provide better percutaneous delivery than the drug solution which was proved by the tape stripping method. As, PLGA is claimed to possess a high ability in penetration of cell membranes (Arafa et al., 2020). On the other hand, Lapteva et al. (Lapteva et al., 2014) adopted an unusual idea by using MPEG-dihexPLA diblock copolymer polymeric micelles and achieved increased aqueous solubility of the drug by 518 folds and deeper skin penetration with deposition in wrinkles and corneocyte clusters.

In a novel approach, Singka et al. (Singka et al., 2010) prepared a polymeric nanogel loaded with methotrexate. Methotrexate, a potent cytotoxic agent, is used as a second-line treatment option in cases of severe plaque psoriasis. Its mechanism of action involves the suppression of the inflammatory mediator PGE2 (prostaglandin E2) and is marketed as systemic products whose use is limited in spite of its efficacy due to its major side effects. Using the drug in a topical formulation may be the solution to this controversy. In this study, nanogels were prepared using polyNIPAM-co-BA; produced by the copolymerization of NIPAM (N-isopropylacrylamide, a biodegradable polymer with low critical solution temp. ∼32 to 34 °C) with butylaccrylate (BA), a nonionic monomer. The point of this combination was to make a thermo-responsive nanogel. The *ex-vivo* results showed good permeation results on the application of the nanogel with or without Na_2_Co_3_. However, Na_2_Co_3_ was useful in reducing the breakthrough time, it also had a significantly positive effect on the reduction of PGE2 with a good safety profile.

Pischon et al. (2017) used a mouse model of psoriasis to explore the pros and cons of targeting psoriatic skin by a fluorescent-labeled dendritic core multishell drug delivery system consisting of the polymer, PG10000(–NH2)_0.7_ (C18mPEG6)_1.0_. *In vivo* results showed consistent accumulation of the dendrimers in the stratum corneum with no permeation to the deeper viable skin layers, high stability and minimum degradation rate over time in both healthy and psoriatic mouse models. This indicated ability of the dendrimers to deliver the drug in spite of the hyperkeratinization associated with psoriasis.

Natural plant extracts also had a good share of advanced colloidal formulations research work, often combined with other chemical antipsoriatic agents. For example, Sun et al. (Sun et al., 2016) used an imiquimod-induced psoriasis-like mouse model and an HaCaT cell-line (cultured human kerstinocyte cells) to demonstrate both *in vitro* and *in vivo* effects of incorporating curcumin, an ingredient of turmeric, that exhibits a good antipsoriatic activity by inhibition of the proinflammatory cytokine, Interleukin, in PLGA nanoparticles hydrogel. The encapsulation of curcumin had significantly improved its biological activity in terms of HaCaT cells apoptosis and PASI score (psoriasis area severity index) that is, better *in vitro* and *in vivo* activity.

Curcumin was also the drug of choice for Mao et al. (Mao et al., 2017) who promoted the penetration of this hydrophobic drug to the skin layers using a novel amphiliphilic graft polymer, RRR-tocopheryl succinate-polylysine (VES-g-PLL), to collect positively charged tiny nanoparticles with a particle size of 24.4 nm. Nanoparticles were then incorporated into a silk fibroin hydrogel and tested for their antipsoriatic efficacy on imiquimod mouse models in comparison to a plain curcumin gel. The results were assessed by the PASI score, histopathological analysis, cytokine inhibition, and angiogenesis inhibition. The encapsulation of curcumin created a sustained release while keeping the skin well hydrated by the gel, which created an occlusive effect and hence promoted its antipsoriatic efficacy.

### 2.2. Metallic nanoparticles

Metallic nanomaterials have been a cause of scientists’ admiration for many decades but they particularly gained the interest of researchers in the past few years in various fields of delivery of therapeutic agents, mainly in the treatment of malignant tumors. Moreover, these particles were shown to be promising in dermatological diseases’ management as well due to their anti-inflammatory effect on the skin (Crisan et al., 2016). Thus, they are widely studied nowadays as skincare products to be used in both dermatology and cosmetology fields as well, owing to their effect as antibacterial agents, antifungal agents, anti-skin cancer, UV radiation filters, targeting agents and high skin penetrating properties as well (Niska et al., 2017). There are various types of metallic nanoparticles synthesized using different elements such as gold, silver, platinum, zirconium, copper, palladium, iron, selenium and strontium, with numerous arrangements such as nanopores, nanotubes, nanorods, nanoclusters, and nanostars (Shankar et al., 2016; Ali et al., 2017; Crisan et al., 2016). One of the major advantages of metallic particles is the ability to be synthesized either chemically or from organic materials, in a process referred to as green synthesis or biosynthesis, which makes this approach not only cost-effective but environmentally safe as well.

The synthesis of silver and gold nanoparticles usually involves using natural materials as plants in a reduction reaction between a gold cation, usually in the form of gold chloride, a reducing agent and a stabilizer; the most commonly used plants in this process include Neem (*Azadirachia indica*), Aloe vera, *Camellia sinensis*, *Catharanthus roseus, Geranium leaf, Datura metel, Cinnamomum camphora* and lemongrass (*cymbogon* sp.) (MIttal et al., 2013; Arafa et al., 2018).

Gold nanoparticles in particular are becoming a popular drug delivery platform owing to their advantageous characteristics that are perfectly suitable for topical drug delivery; ease of functionalization, large surface area compared to volume and small particle size in addition to the anti-inflammatory effect they exert themselves which creates a synergistic effect when loaded with anti-inflammatory agents (David et al., 2014; Bessar et al., 2016). The magnificent properties of these nanoparticles are referred to surface plasmon resonance properties; which is explained by El-Sayed et al. (Eustis & El-Sayed et al., 2006) as the oscillating movement of surface electrons around the noble metal particles when exposed to radiation with a wavelength larger than the diameter of the particle itself. Thus, creating a change in the electric field surrounding the particle and creating a massive change in the particle properties. Hence, different sizes. Therefore, changing gold nanoparticles size, shape, solvent or surrounding material will lead to a massive change in their properties such as color change. Gold nanoparticles have different colors that range from orange, yellow, red or blue according to their shape and size. Hence, can be used for different applications including drug delivery. Silver nanoparticles also do offer advantageous characteristics for drug delivery such as targeting, improving solubility and stability in addition to enhancing efficacy and hindering side effects by reducing the required drug dose (Rehman et al., 2012).

In the following section, we will discuss in short the approaches that were explored by different researchers using the magnificent properties of these metallic nanoparticles, especially their very small size, in the management of plaque psoriasis by conjugation with natural or synthetic anti-inflammatory agents. So far, scientists have explored the use of gold, silver and platinum nanoparticles for the topical management of psoriasis. The recent approaches that used these metallic nanoparticles for topical treatment in psoriasis are summarized in (Table 3).

**Table 3.** Recent approaches utilizing metallic nanoparticles in management of chronic psoriasis.

Metallic nanoparticle	P.S range	Active ingredient	Method of preparation	Conclusion
Gold	13–52 nm	Corneal cherry (*Cornus mas*)extract	Green synthesis	Nanoparticles specifically target macrophages in psoriatic skin plaques (Crisan et al., 2018). No significant cytotoxic effects on culture cell lines.
	2–24 nm			TNF-α decreased production and IL-6 increased production in culture cell line (Perde-Schrepler et al., 2016) Lower cytotoxicity than chemically synthesized citrate-coated gold nanoparticles.
	10–15 nm	Short interfering RNAs	Chemical synthesis	Efficient suppression of gene expression and T cell production (Nemati et al., 2017). No toxicity on skin cells.
	4–5 nm	Methotrexate	Chemical synthesis	Efficient cell growth inhibition when compared to drug alone and deeper skin penetration. No reported toxicity on skin cells (Bessar et al., 2016).
	11–90 nm	A natural extract of berries; cyaniding 3-O-glucoside and cyaniding 3-O-sambuboside	Green synthesis	Anti-inflammatory effect of cyaniding 3-O-sambuboside > cyaniding 3-O-glucoside > hydrocortisone (Crisan et al., 2013) Immunohistological examination showed no cytotoxicity both *in vitro* and *in vivo*.
Silver	9–82 nm	Corneal cherry (*Cornus mas*)extract	Green synthesis	Nanoparticles specifically target macrophages in psoriatic skin plaques (Crisan et al., 2018). No significant cytotoxic effects on culture cell lines.
	20–80 nm	A natural extract of berries rich in polyphenols and anthocyanins	Green synthesis	Proved to decrease cytokine production both *in vitro* and *in vivo* (David et al., 2014). Better anti-inflammatory effect than hydrocortisone. Cytotoxicity reported.
Platinum	1.7–3.1 nm	No added therapeutic agent	Chemical synthesis	Inhibition of a major signaling pathway of inflammation (Rehman et al., 2012) No vital cytotoxic effects reported.

Regarding polyphenols used with metallic nanoparticles. Polyphenols are natural compounds that are available in many diet products, they exert an antioxidant and anti-inflammatory activity that has shown a useful effect in the treatment of psoriatic lesions by minimizing the activity of enzymes responsible for the production of ROS (reactive oxygen species) (Hussain et al., 2016).

Crisan et al. (Crisan et al., 2013) loaded a natural extract of berries rich in polyphenols into gold nanoparticles, synthesized by the reduction of gold ions in HAuCl4. The particles showed a small size range (11–90 nm). The anti-inflammatory effect of the particles (in terms of production of cytokines, cell morphology, and viability) was assessed *in vitro* on an HaCaT cell-line after exposure to UVB radiation and compared to the berry extract alone. Cells treated with gold nanoparticles before exposure to irradiation showed a significant reduction in cytokine production with no significant cytotoxicity in comparison to those treated with berry extract only which may be attributed to the synergistic effect exerted by the anti-inflammatory properties of the gold nanoparticles themselves. The team also performed a clinical study on psoriasis patients using different moisturizing creams; containing anthocyanin cyanidin 3-O-glucoside gold nanoparticles, cyanidin 3-O-sambubioside gold nanoparticles, and hydrocortisone cream. The results confirmed that both gold nanoparticle creams had a better anti-inflammatory effect on treated subjects than hydrocortisone cream. The authors referred this to the longer residence of nanomaterials on the epidermal skin layer which is considered the major site of action in the management of psoriatic inflammatory hyperproliferation. Another research team, David et al., (David et al., 2014) incorporated the berry extracts in silver nanoparticles instead to obtain a particle size ranging from 20 to 80 nm and a significantly reduced production of IL-1 α compared to the control cells. However, there was an observed cytotoxic effect of Ag nanoparticles on cells in terms of loss of the epithelial shape. Despite that, it was reported in the literature that the use of natural materials such as epicatechin and *Albizia adianthifolia* leaf, as a source of an –OH functional group on the nanoparticles, had no cytotoxic effect on HaCat cell line resembling human peripheral lymphocytes.

Furthermore, Bessar and his team (Bessar et al., 2016) loaded Methotrexate on gold nanoparticles for the aim of the treatment of plaque psoriasis. This approach of methotrexate conjugation to gold nanoparticles has been used in cancer treatment but was considered a novelty in the field of dermatology. Gold nanoparticles were synthesized chemically and functionalized with 3-mercaptopropanesulphonate and 2-Diethylaminoethanethiol hydrochloride then loaded with methotrexate. *In vitro* tests recommended that the methotrexate loaded gold nanoparticles had no toxicity on skin cells while giving efficient cell growth inhibition, while *in vivo* tests suggested deeper skin penetration of the nanoparticles to the dermis. The authors considered this an advantage in the treatment of psoriasis as the psoriatic inflammatory mediators exist in the dermis.

One of the most interesting studies in this topic was done by Nemati et al. (Nemati et al., 2017), who adopted the combination of the two novel approaches; gene therapy and gold nanoparticles delivery for the treatment of plaque psoriasis by application of siRNAs (short interfering RNAs) for gene regulation in animal models. The team used an EGFR-SNA-NCs (epidermal growth factor receptor spherical nucleic acid nanoparticle conjugate) as a carrier system to deliver siRNAs safely to the cell nucleus, thus escaping hydrolysis by nucleases and cellular barriers. The gold nanoparticles were synthesized by a reduction reaction using HAuCl4 and sodium citrate then siRNAs were conjugated to the pre-synthesized gold nanoparticles. The obtained particle size range was 10–15 nm. The nanoparticles showed no cytotoxicity with a significant downregulation of gene expression ranging between 21.76 and 91.4%. Additionally, the *in vivo* experiments showed no toxicity on mice skin neither in terms of erythema nor scaling or thickening. Hence, proving to be a promising approach with a high potential for further research in dermatological autoimmune diseases’ management.

*Cornus mas* (corneal cherry) is a medicinal plant of high value due to its various medicinal uses, proven antioxidant activity, anticancer, and anti-inflammatory effects (Hosseinpour-Jaghdani et al., 2017). Perde-Schrepler and his fellow researchers (Perde-Schrepler et al., 2016) extracted the crushed fruits using distilled water and vacuum and collected an extract *rich in polyphenols and anthocyanins*. They incorporated the extract in gold nanoparticles, synthesized by a green reduction method, to produce spherical nanoparticles with a size range of 2 to 24 nm with maximum plasmon absorption and stability in physiological solution. The nanoparticles’ effect was assessed in terms of cytotoxicity, ROS production and the production of proinflammatory mediators after exposure to UVB irradiation stimulus.

Crisan D and his team (Crisan, 2016) took this study to a new level by using the cornus mas extract in both silver and gold nanoparticles and explored their effects *in vitro* on activated macrophages to mimic the psoriatic skin inflammation, *ex-vivo* on inflamed skin biopsies collected from psoriasis’ patients and finally *in vivo* as a clinical study on 24 subjects suffering from chronic stationary plaque psoriasis. The silver and gold nanoparticles were synthesized by an eco-friendly method and incorporated in an ointment. Both types of nanoparticles showed a significant suppression in the production of proinflamamtory cytokines (IL-12 and TNF-α) compared to the control group during immunohistochemistry study. The clinical study confirmed the previous results on human psoriatic plaques, showing that the metallic nanoparticles specifically target skin macrophages and suppress the production of inflammatory mediators leading to a significant improvement in the therapeutic outcome.

On the other hand, Rehman et al. (Rehman et al., 2012) studied the use of platinum nanoparticles as an anti-inflammatory agent and found out that it has a down regulatory effect on the NF*_K_*B signaling pathway, which was stated by Zolotarenko et al. (Zolotarenko et al., 2017) to be one of the core transcriptional regulators involved in psoriatic inflammation. The research team used a reduction reaction using citrate and H_2_PtCl_6_ (chloroplatinic acid) to prepare platinum nanoparticles and proved the ability of the aforementioned nanoparticles to inhibit a major signaling pathway of inflammation by inhibition of the phosphorylation of Akt and ERK1/2 and consequently the inhibition of NF*_K_*B production while showing no cytotoxicity on cell culture line.

Metallic nanoparticles were accused of their cytotoxicity to epidermal keratinocytes and hair follicles’ stem cells because of producing reactive oxygen species (ROS), metal ions are also thought to cause skin irritation (Bessar et al., 2016; Crisan et al., 2018; Wang et al., 2018). They were also claimed to facilitate skin penetration leading to systemic absorption and possible undesirable effects especially in the case of psoriasis where the barrier function of the skin is disrupted. However, the point of whether metallic nanoparticles pass the skin barrier or remain retained in the skin layers was thoroughly studied by many researchers and is controversial in the literature (Wang et al., 2018). It was proven that skin penetration or retention is actually dependant on particle size, functional groups, and particle shape. Niska et al. state that very small-sized particles (<4 nm) can cross the skin barrier while small-sized particles (4–20 nm) can cross damaged skin but not intact one. While, particles with size >45 nm can neither penetrate intact or damaged skin (Niska et al., 2017). In agreement, Miquel-Jeanjean and his peers tested the penetration of TiO2 nanoparticles in psoriatic and normal skin and found out that there was no difference between both skin types as in both cases nanoparticles were retained in the epidermis and did not reach the dermis layer (Miquel-Jeanjean et al., 2012).

Metal nanoparticles pass the skin barrier through intercellular, intracellular and transappendegeal routes. But, the altered psoriatic skin condition should be taken into consideration due to the disrupted tight junction between cells (Wang et al., 2018). Despite, using metal nanoparticles for treatment of psoriasis is worth studying because of the antiinflammatory and antimitotic properties they exert (Crisan et al., 2013). Gold nanoparticles synthesized by green methods have low cytotoxicity (Perde-Schrepler et al., 2016).

The previously discussed articles studied cytotoxicity and observed that no significant cytotoxic effects appeared on using gold and platinum particles on psoriatic skin (Rehman et al., 2012; Crisan et al., 2013; Bessar et al., 2016; Perde-Schrepler et al., 2016; Nemati et al., 2017; Crisan et al., 2018). Perde-schepler et al. specified that gold nanoparticles synthesized by green synthesis exerted less cytotoxic effect than those synthesized chemically. While silver nanoparticles reported cytotoxicity in one study (Crisan et al., 2018) and reported no cytotoxicity in another (David et al., 2014) indicating the need for further investigation.

### 2.3. Lipidic nanoparticles

Lipid-based nanoparticles are a widely spread type of nanoparticles that were broadly studied by scientists since the first attempts to use nano-sized carriers in the field of drug delivery. They have the advantage of being a natural carrier that can be available and cost-effective. The classical examples of lipid-based nanocarriers include liposomes, Poly-amphiphiles (such as liposomes and niosomes), solid-lipid nanoparticles (SLNs) and nano-structured lipid carriers (NLCs) as well as nanoemulsions (Puri et al., 2009; Bhatia, 2016). Scientists used these types of drug carriers extensively to target psoriatic plaques topically. Some of these approaches were previously summarized in more than one review article as those reviewed by Dubey et al., Katare et al., Sala et al. and Pradhan et al (Katare et al., 2010; Pradhan et al., 2013; Sala et al., 2016; Dubey et al., 2017; Sala et al., 2018). we will discuss briefly in the following paragraphs most of the recent approaches that were adopted in this field for the topical management of psoriatic plaques, their synthesis techniques, and research findings.

#### 2.3.1. Particulate lipidic systems

Solid-lipid nanoparticles (SLNs) and nanostructured lipid carriers (NLCs) are two types of drug carriers, together referred to as particulate lipidic systems, that were used since the nineties of the previous century. SLNs are composed of a solid lipid core particulate system dispersed in a liquid lipidic phase, it usually carries hydrophobic drugs that are dispersed within the core of the lipid particle. Nanostructured lipid carriers; usually considered the successor of SLNs, are also composed of a hybrid between solid and liquid lipid systems however they do not have the perfect crystal structures of the SLNs. This imperfect structure allows for better drug encapsulation and less drug leakage (Puri et al., 2009). Another particulate system is the lipospheres, which constitutes a solid lipid core surrounded by a single phospholipid layer. The main advantage of this system is the ability to carry both hydrophilic and hydrophobic drugs and not restricted to hydrophobic drugs as SLNs and NLCs (Dudala et al., 2014). All the aforementioned systems were used as a carrier for antipsoriatic drugs in many research articles; Puglia et al. (Puglia et al., 2012) tackled the use of particulate lipidic systems as a delivery system for topical use in both cosmetics and the management of dermatological diseases as psoriasis. We endeavored to summarize the previously published work in Table 4.

**Table 4.** A summary of lipidic nanosystems for topical treatment of psoriasis in the past few years.

Delivery system	Production technique	Drug	Findings
Particulate systems			
SLNs	Modified emulsification and low temperature solidification method	Tacrolimus	Higher skin penetration and retention than marketed ointment (Ruihua, 2012).
	Thin film hydration	Erlotinib + IL α 36SiRNA	Decreased epidermal hyperplasia (Boakye et al., 2017). Decreased infiltration of inflammatory cytokines.
	Hot ultrasonication	Methotrexate + Etanercept	Decreased transdermal permeation (Ferreira et al., 2017).
	Solvent injection	Mometasone Furoate	Higher skin deposition than commercial cream (Madan et al., 2014).
	Solvent injection	Betamethasone 17-valerate	Controlled release (Zhang and Smith, 2011). Drug retention in epidermis.
	Pre-emulsion ultrasonication	Dithranol	Double drug localization than the commercial cream (Zhang and Smith, 2011).
NLCs	Hot emulsion sonication	Thymol	Improved healing of psoriatic plaques in mouse models (Pivetta et al., 2018).
	Microemulsion	Mometasone Furoate	Disappearance of parakeratosis^a^ (Kaur, Sharma and Bedi, 2018). Increased skin deposition 2.5 times than marketed product.
	High-shear homogenization	Methotrexate	Enhanced skin penetration (Pinto et al., 2014).
	Microemulsion technique	Clobetasol propionate	Increased drug accumulation in the stratum corneum (Silva et al., 2012).
	Hot homogenization	Tacrolimus	Higher penetration than commercial product (Nam et al., 2011).
	Thin film hydration	Calcipotriol + Methotrexate	Suppressed skin permeation of calcipotriol only (Fang, 2010). Lower skin irritation.
	A modified microemulsion technique	Fluticasone propionate	Improved drug encapsulation and formulation physicochemical stability (Doktorovová et al., 2010).
A comparative study between SLNs and NLCs	Hot melt homogenization method	Cyclosporin & calcipotriol	Both particulate systems show deeper penetration (Arora et al., 2017). NLCs exhibited higher efficacy than SLNs and commercial gel.
	Solvent diffusion technique	Capsaicin	Both particulate systems minimize skin irritation (Agrawal et al., 2015). NLCs show better stratum corneum permeation and retention than both SLNs and plain drug.
	Microemulsification	Tretinoin	Higher efficacy and biocompatibility than the commercial product containing Tretinoin (Raza et al., 2013). Particulate lipidic systems show higher photostability and skin permeation than vesicular systems.
Lipospheres	Modified emulsion-based method	Thymoquinone	Improved histopathological and psoriatic features of psoriatic skin both *in vivo* and *in vitro* (Jain et al., 2017). Decreased TNF-α, IL-2, IL-6 and IL-1β.
	Modified emulsion-based method	Tacrolimus + curcumin	Improved histopathological and psoriatic features of psoriatic skin *in vivo* (Jain et al., 2016). Decreased TNF-α, IL-17,IL-22.
Liquid crystals	Lipid melting, mixing with the aqueous phase and sonication	siRNAs	Gene downregulation GAPDH suppression (Vicentini et al., 2013).
		siRNAs	Reduction of IL-1α and IL-6 production (Depieri et al., 2016).
Vesicular systems			
Liposomes	Thin film hydration	Fusidic acid	A more stable formulation with a higher efficacy (Wadhwa et al., 2016).
	Thin film hydration	Tretinoin	Particulate lipidic systems show higher photostability and skin permeation than vesicular systems (Raza et al., 2013).
	Thin film hydration	Calcipotriol	A smaller particles size (<100 nm) and a unilamellar structure which promoted skin penetration and drug deposition (Knudsen et al., 2012).
	Thin film hydration	Methotrexate	Increased skin permeability (Srisuk et al., 2012).
	Thin film hydration	Tretinoin	Higher skin deposition (Manconi et al., 2011).
Niosomes	Thin film hydration	Anthocyanins	Prolonged antiinfalmmatory effect with no cytotoxicity (Manconi et al., 2011).
Ethosomes	Modified injection method	Psoralen	Improved permeation and skin deposition (Zhang et al., 2014).
	The cold method	Tretinoin	Particulate lipidic systems show higher photostability and skin permeation than vesicular systems (Zhang et al., 2014).
	Thin film hydration	5-aminolevulinic acid	Enhanced accumulation in both normal and hyperproliferative skin (Fang et al., 2009). Higher penetration depth Reduced TNF-α expression
A comparative study between liposomes and niosomes	Thin film hydration	Dithranol	Both vesicles showed better skin permeation both *in vitro* and *in vivo* (Agarwal et al., 2001).
A comparative study between emulsomes, liposomes and niosomes	Thin film hydration	Capsaicin	Emulsomes showed to be a convenient carrier achieving higher penetration and retention (R. Gupta et al., 2016).
SECosomes^b^	Solvent evaporation method	RNAi	Downregulation of the psoriatic genetic marker (human beta-defensin 2) (Desmet et al., 2016).
Emulsion systems			
Microemulsion	Titration	Protopanaxdiol	Higher *in vivo* and *in vitro* skin deposition (Kim et al., 2018).
	Titration	Methotrexate	Increased epidermal drug accumulation with lower systemic absorption, that is, improved efficacy and safety (Amarji et al., 2016).
	Aqueous phase titration	Betamethasone dipropionate + salicylic acid	Improved and sustained anti-inflammatory activity and higher skin penetration (Baboota et al., 2011).
	Shaking mixtures of the components of the microemulsion with the calculated ratios	Tacrolimus	Superior bioavailability (Baboota et al., 2011).
Nanoemulsion	High shear homogenization	Cyclosporin	Increased drug permeability *in vitro* and superior skin hydration on human volunteers (Musa et al., 2017).
	Spontaneous emulsification	Clobitasol propionate + calcipotriol	Better uptake by stratum coreum *in vitro* (Kaur et al., 2017). Better antipsoriatic skin activity *in vivo*.

^a^A type of cell keratinization where the nuclei are retained in the stratum corneum. ^b^A liposomal carrier developed by the authors.

The most commonly used method for the preparation of SLNs is high-pressure homogenization; including hot and cold homogenization methods.

*Hot homogenization,* is the dispersion of the drug in a solubilized lipid followed by the emulsification of this lipid in a hot aqueous surfactant system then, homogenizing the emulsion to collect the crystallized SLNs (Müller et al., 2000).

*Cold homogenization,* is the crushing of a cooled lipid after dispersing the drug in it while the lipid is solubilized then, dispersing the lipid in a cold surfactant (e.g. poloxamers and compritol) to get a pre-suspension which is further homogenized then SLNs are collected (Müller et al., 2000; Gupta, Kesarla, et al., 2017).

*Microemulsion* method is In this method a microemulsion is formed of the melted lipid with the drug, water, a surfactant, and a co-surfactant under non-vigorous stirring (Müller et al., 2000).

Hot homogenization technique usually produces smaller particle sizes. However, it may accelerate the drug or carrier hydrolysis. Therefore, cold homogenization is preferred in the case of thermolabile constituents but it does not abolish heat exposure totally as heat is also involved in the last step of the process as well as the disadvantage of producing larger particle size and size distribution (Mukherjee et al., 2009). While microemulsion is the favorable production method of SLNs on a large scale actually it is a favorable technique particularly on taking these particles to the mass production level.

There are also other techniques to prepare SLNS including emulsification-ultrasonication, solvent emulsification diffusion/evaporation, Film-ultrasonication method, hot-melt extrusion, and supercritical fluid methods.

Likewise, NLCs are mainly prepared by high-pressure homogenization and microemulsion techniques while the other aforementioned techniques may be used as well (Salvi & Pawar, 2019). On the other hand, lipospheres are prepared by the melting method, co-solvent method or spray drying, spray congealing, multiple microemulsion and supercritical fluid methods (Dudala et al., 2014).

Liquid crystals are a state of matter that combined fluidity of liquids with ordered crystal shape of solids and one of its advantages is having two melting points using a combination between a cationic polymer or lipid and certain types of lipids (e.g. monoolein and oleic acid) (Andrienko, 2018). The interest in gene therapy as an effective way of eliminating the psoriatic plaques from their roots of origin has been increasingly evolving, especially in severe cases of psoriasis where the management using anti-inflammatory agents and immunosuppressants is not sufficient to deter the disease. In two different research articles both Vicentini and Depieri (Vicentini et al., 2013; Depieri et al., 2016) with their peers used siRNAs to silence particular genes responsible for the magnified skin cell replication caused by the autoimmune response involved in psoriasis. They achieved diminished protein production of these cells by fabricating a liquid crystalline nanodispersion which effectively incorporates macromolecules and bypasses the barrier of the stratum corneum to reach its molecular target successfully. Vincentini (Vicentini et al., 2013) and his fellows have used the lipids monoolein and oleic acid along with the cationic polymer PEI (polyethyleneimine) or the cationic lipid OAM (oleylamine) in hexagonal-shaped liquid crystals dispersed in an aqueous phase for siRNAs delivery in the form of topical preparation. This achieved good *in vivo* and *in vitro* results and was able, by gene downregulation, to suppress the GAPDH protein with no skin irritation. Depieri (Depieri et al., 2016) and his fellows have optimized the previous formula by reducing PEI concentration to prevent cytotoxicity. They also assessed their nanodispersion’s efficacy *in vitro* both on pig’s ears’ skin and on reconstructed human epidermal skin cell lines by measuring the psoriatic markers: IL-1α and IL-6. Where both markers’ production was shown to be significantly reduced.

#### 2.3.2. Vesicular lipidic systems

In general, lipid-based vesicular systems are defined as a concentric bilayer(s) an amphiphilic substance that is self-assembled in an aqueous solution. With their various types, they are considered are one of the most common nanosystems that have been studied for the purpose of drug delivery. They introduced various advantages in the field of drug delivery; attributed to the ability of these systems to localize the drug in their core or in the bilayer which ensures loading drugs of different natures, improved bioavailability, targeting of certain organs, stability and reduced toxicity (Jain et al., 2014).

Liposomes are the most extensively reviewed system of this type; they consist of a natural or synthetic bilayer/s of phospholipids enclosing an aqueous core and cholesterol as a fluidity modifier (Bansal et al., 2012; Buchiraju et al., 2013; Jain et al., 2014). Liposomes, in particular, were widely studied for the purpose of drug delivery for the management of inflammatory conditions, including psoriasis (Rahman et al., 2016).

Other forms of these bilayered structures have emerged, with the aim to tailor and improve delivery characteristics in the favor of achieving efficacy and safety for the management of various illnesses. Different materials were used to shape these bilayers and their cores. Each of these systems has its pros and cons. Tailoring the drug delivery system according to the aim of work, drug characteristics and the physiology of the target site is recommended to achieve the desired outcomes. Table 5 summarizes most of the types of vesicular lipidic systems that have appeared in the past few years and their basic constructing materials. On reviewing the literature, we found out that Psoriasis was a target disease for many investigators who used lipidic vesicular systems as drug carriers to achieve efficacy and safety. Those reviewed approaches are also summarized in Table 4.

**Table 5.** A summary of vesicular lipid drug delivery systems that have been invented by formulators in the past years.

Vesicular system	Definition
Liposomes	Natural or synthetic bilayer/s of phospholipids and cholesterol as a fluidity modifier (Bansal et al., 2012; Jain et al., 2014).
Niosomes	One or more bilayers consisting of amphiphilic nonionic surfactants instead of phospholipids (Bansal et al., 2012; Jain et al., 2014).
Trasferosomes	Flexible and deformable liposomes (Bansal et al., 2012; Jain et al., 2014).
Aquasomes	A solid ceramic core with a carbohydrate coat (Jain et al., 2014).
Colloidosomes	Spherical structures with a hollow core and a coat of particles that are formed by coagulation at the interface of emulsion droplets (Jain et al., 2014).
Cubosomes	Cubic shaped vesicles (Jain et al., 2014).
Sphingosomes	Liposomes formed of sphingolipids instead of phospholipids (Jain et al., 2014).
Ufasomes	Unsaturated fatty acids forming bilayered structure that attach to the surface of the skin (Patel et al., 2011).
Cryptosmes	Vesicular structures with a coat of phosphatidyl choline and phosphatidyl ethanolamine (U. et al., 2019).
Discomes	Disc-shaped structures similar to niosomes (Uchegbu et al., 1992).
Emulsomes	Drug nano-carriers with a solid lipid core and a phospholipid bilayer surface (Zhou and Chen, 2015).
Enzymosomes	Liposomes with a functional group of one or more enzyme (S. et al., 2017).
Genosomes	Vesicles that consists of genetic material and positively charged lipids (Tadwee et al., 2011).
Ethosomes	Liposomes that contain: ethanol, water and a penetration enhancer (Tadwee et al., 2011).
Photosomes	A liposome that incorporates photolysase enzyme (Tadwee et al., 2011).
Virosomes	Phospholipid vesicles with virus-extracted proteins (Tadwee et al., 2011).
Vesosomes	Multivesicular structures synthesized using ethanol and certain types of phospholipids (Tadwee et al., 2011).
Proteasomes	Lipid vesicles with protease enzymes (Tanaka, 2009).
Herbosomes	Liposome-like structures incorporating phytochemicals (Anwana, 2015).
Layerosomes	Phospholipid bilayer vesicles with a surface of polyelectrolytes (Agnihotri et al., 2004).
Pharmacosomes	Very fine particles consisting of covalently linked drug and lipids (Bansal et al., 2012).

Being an ancient type of vesicles, liposomes have various methods of preparation according to many aspects: their size, number of bilayers and stealth. Generally, drug loading to the phospholipid is either active or passive (Akbarzadeh et al., 2013). Passive drug loading is the loading of the drug during liposomal preparation via mechanical dispersion

(sonication, extrusion, freeze-thawing, lipid film hydration, micro emulsification, extrusion or drying-reconstitution), solvent dispersion (ether injection, ethanol injection or reverse-phase evaporation) and detergent removal (dilution, chromatography or dialysis). Active drug loading is a technique applied for hydrophilic drugs, in which the drug is loaded after the synthesis of the vesicle by an ionic conjugation using PH alteration. The same methods can be applied to nonionic surfactants for the purpose of preparing noisomes (Moghassemi & Hadjizadeh, 2014). In addition, the bubble method which has the advantage of avoiding the use of organic solvents and the heating method.

#### 2.3.3. Micro/nano emulsion systems

It is axiomatic that an emulsion is a dispersion of minute droplets of one liquid in another, both being insoluble. Micro and nanoemulsion systems incorporate droplets with a minute diameter, in the nano range. Owing to their many advantages, these types of colloidal systems gained the interest of researchers in various fields, including of course the pharmaceutical industry field. These systems offer better stability and resistance to coalescence, a clear appearance, higher flexibility regarding rheological properties of the final dosage form, as well as a potential bioavailability enhancing system for lipophilic drugs (Mason et al., 2006; McClemets, 2012).

On reviewing literature tackling this type of dosage form, we found out that there is widespread confusion between both terms, microemulsion, and nanoemulsion. Unexpectedly, as described by David Julian (McClemets, 2012) in his review article, the difference between both terms is not related to the internal phase droplet size but pertains to the thermodynamic stability of the colloidal system as a whole, described as follows:

A microemulsion is a system with a droplet size within the nano range, that is, a colloidal system, containing a surfactant and co-surfactant to achieve enhanced absorption, thermodynamic stability, permeability and solubilizing capacity particularly for hydrophobic agents (Gupta, Sharma, et al., 2017; Viswanathan et al., 2017). While a nanoemulsion is a colloidal particulate system composed of oil, water, and a surfactant and is thermodynamically metastable (Javadzadeh & Azharshekoufeh Bahari, 2017). A microemulsion is thermodynamically stable when kept in certain conditions while nanoemulsion is thermodynamically unstable, but it can be preserved in a metastable state when sufficient energy barrier is maintained between both phases (McClements, 2012). The particle size range that distinguishes microemulsion from nanoemulsion is not unified in different articles. However, it was agreed that the microemulsion particle size range is smaller which gives it a translucent appearance. Table 4 summarizes emulsion nanosystems that were recently used to treat psoriatic plaques topically.

Microemulsion preparation usually depends on preliminary trials to determine the optimum ratio between aqueous phase, oily phase, surfactant and co-surfactant with the ternary phase diagram being the conventional approach to determine these ratios (Rosano et al., 1987). Usually, a coarse emulsion is initially obtained by adding the dispersed and continuous phases in the presence of a surfactant under continuous mixing and the amount of co-surfactant is titrated to obtain a clear microemulsion. Hence, the formulation of nanoemulsion resembles that of the ordinary emulsion while minimizing the droplet size using either high energy emulsification (ultrasonic emulsification, high-pressure homogenization, microfluidization, high energy stirring or membrane emulsification) or low energy emulsification (spontaneous emulsification, phase inversion temperature or emulsion inversion point) techniques (Jaiswal et al., 2015). Another emulsion-based nanosystem is the nanoemulgel, which consists of a nanoemulsion that is further incorporated in a gel using an appropriate polymer. It is thought to be favorable in dermal applications and for the incorporation of antipsoriatic agents for topical application (Anand et al., 2019).

Solid nanoparticles, for example, SLNPs and NLCS are specified to be more retained in skin than deformable nanoparticles such as vesicular systems, for example, liposomes and transferrosomes which cross the skin barrier more readily so, they tend to be used for transdermal purposes (Knorr et al., 2019). However, the extent of particles’ penetration is subjective according to different factors such as particle size, charge and skin condition; including skin integrity or thickening. As previously mentioned, according to researchers’ experimental trials many vesicular systems showed satisfactory results in psoriatic plaques control.

### 2.4. Hybrid nanosystems

To cope with all requirements of a perfect dosage form for the management of psoriasis in the most convenient way for the patient, some researchers have adopted novel ideas that merged two different types of nanomaterials, particularly polymers and lipids to benefit from the advantages of both.

Researchers combined microemulsion with polymers to enhance the permeation of antipsoriatic agents. Jitendra Behera (Behera et al., 2010) and Wan (Wan et al., 2017a) along with their coworkers adopted this idea. The former synthesized a microemulsion formula from soybean oil, egg phosphatidylcholine, ethanol, and an aqueous phase and used chitosan as a coating material to deliver methoxsalen (a natural material of a plant origin which may cause cell cytotoxicity by DNA synthesis inhibition) to the skin. The results showed better skin accumulation compared to the microemulsion alone. While, the latter used a polymer-based microemulsion as a carrier system for the immunosuppressing agent, tacrolimus. The microemulsion consisted of Labrafil M, Labrasol/TPGS and Transcutol P as the cosurfactant. TPGS (Tocopheryl Polyethylene Glycol 1000 Succinate) was their polymer of choice along with labrasol to act as the surfactant. The formula was assessed *in vitro* on HaCaT cell line and proved better skin permeation in comparison to the marketed product of Tacrolimus with antiproliferative effect, PASI score, histopathological features, and drug’s deposition. Furthermore, the blank formula showed an antinflammatory effect on psoriatic skin cells which was an adjuvant to the immunosuppressive action of Tacrolimus. All the previous data suggested that this carrier system is potentially very appropriate for topical targeting of psoriatic plaques (Wan et al., 2017b).

Another research team represented by Ridolfi (Ridolfi et al., 2012) used SLNs and chitosan for the delivery of tretinoin to keratinocytes and compared their system to SLNs as a stand-alone nanosystem. The hybrid system showed higher encapsulation efficacy and less cytotoxicity. Moreover, it had an antibacterial action, as their study involved acne targeting as well. The previous results proved that the hybrid system allowed more efficient and safer drug use for psoriatic lesions.

Alternatively, Desai et al. (Desai et al., 2013), used capsaicin, the natural extract of capsicum, in a combination with anti-TNFα siRNA to target psoriasis. The combination was encapsulated in a cationic lipid-polymer hybrid nanocarrier and tested on a psoriasis-like mouse model. The design of the nanocarrier comprised: a PLGA core to incorporate the hydrophobic capsaicin (negatively charged), a lipid layer to retard drug escape from the core with a cyclic polar head group that controls drug release (positively charged) and a positively charged lipid layer with cyclic pyrrolidinium head-group at the interface between the hydrophobic core and hydrophilic PEG shell, hence providing stealth and deeply delivering SiRNA into skin layers. The *in vivo* results (presented in PASI score) demonstrated the synergistic effect of gene and drug delivery into deeper skin layers by the novel nanocarrier.

## 3. Dosage form choice

On writing this article, we have actually reviewed tens of different research experiences performed in different countries to find out that many researchers nowadays do not get involved in using a second dosage form as they prefer to use the colloidal system in its liquid state in their tests whether *in vivo, in vitro* or both. Yet, others do take their formulas to the next step by incorporating them in suitable semisolid dosage forms. For instance, a significant number of researchers chose to synthesize a gel whether a hydrogel, an amphiphilic gel or a chitosan gel (Ahmed, 2015). One smart idea was to use a nanogel as the primary delivery system to incorporate the drug, this idea was executed by Singka and his peers (Singka et al., 2010) to act as a carrier for methotrexate where the hydrogel was composed of N-isopropylacrylamide (NIPAM) and butylacrylate (BA) co-polymer. Others were more conventional and used an ointment or a cream.

We do suggest another type of dosage form, which was not thoroughly investigated by researchers for the purpose of controlling psoriatic plaques, which is the spray. In fact, there are many types of sprays including foam, emulsion, suspension, and film-forming sprays. It actually may be very convenient for the patients as it does not leave a greasy residue on exposed areas as the face and arms which allows the patient to apply the medication day and night and not only before bedtime. Film-forming sprays are readily retained in the stratum corneum, they are fast drying and nonirritating (Kathe & Kathpalia, 2017). Their stability depends on the choice of polymer. Besides, it requires no rubbing for the inflamed areas, which minimizes microbial contamination, and dosage adjustment is more accurate where a metered-dose aerosol may be used (Lulla et al., 2004). This may lead to improved compliance and less frequent dosing. Psomadakis et al. highlighted in their review, the use of some uncommon dosage forms such as the spray and nail lacquer for nail psoriasis (Psomadakis & Han, 2019). The proper choice of the suitable delivery technique in combination with the achieved advances in nanoparticle design, formulation sciences, and improved understanding of particulate carrier systems, skin interactions will undoubtedly lead to significant progress in their topical and transdermal drug delivery applications (Abdel-Mottaleb, 2016; Abdel-Mottaleb & Lamprecht, 2016). Therefore, It is expected during the upcoming years that the advanced technology of drug delivery, using nanotechnology in addition to a suitable dosage form may offer a better quality of life for psoriasis patients, regardless of the disease’s severity, without the need of systemic intervention and its accompanying side effects. Though not curable, psoriasis may be controlled by advanced drug technology.

## 4. Conclusion

Psoriasis is a chronic dermatological disease of underlying autoimmune etiology, unfortunately with no permanent remedy. However, controlling the manifestations of psoriasis significantly improves patients’ quality of lives in order to mimic that of a normal person with none of the psychological burdens associated with the physical appearance of the psoriatic silvery scales. Being one of the greatest accomplishments in science during the past century, nanotechnology has served as a potential solution for controlling psoriasis. Despite the challenges of drug delivery in dermatological conditions in general, including atopic dermatitis, acne, and others, the target is always to achieve maximum possible epidermal penetration and retention with minimum absorption of the drug to the bloodstream to avoid serious side effects. The major challenge regarding psoriasis, in particular, is the thickening of the stratum corneum layer by excessively keratinized skin, making it an even harder barrier to circumvent by different active ingredients.

In fact, many publications have studied this point but still, there is more that can be done. Both polymeric and lipidic nanoparticles have been thoroughly studied for the purpose of Psoriasis management in the past few decades. The emerging interest in metallic nanoparticles in the present era may be useful for this purpose. Owing to their small size and magnificent properties, they may represent a suitable drug carrier for skin penetration and retention. One research experience that significantly highlighted the use of metallic nanoparticles, particularly silver and gold ones. This research, in particular, may be very helpful for other researchers to build on and use this approach using other metals and drugs.

Another rising approach is the hybrid nanosystems since researchers wish to combine the advantages of different types of nanoparticles to get the most suitable drug delivery system for the patient. This approach was not thoroughly investigated for the purpose of topical management of psoriasis and can offer more satisfying results. Limited experiences of hybrid nanoparticles for topical use on psoriatic plaques include one that deserves to be highlighted for further experiments that used liquid crystal nanodispersions of a lipid with a cationic polymer for gene delivery, a type of therapy that relatively cures the disease. These hybrid nanosystems may have the potential to significantly improve the management of these psoriatic plaques.

It is worthy to mention that designing a suitable dosage form that improves patient compliance by reducing the dosing frequency and abolishing a residual oily or gel-like remnant on the skin requires further investigation. Most researchers stick to conventional polymer-based hydrogels, ointments or creams whose residues on the arms and face could be offensive to the patient. Controversially, we suggest a spray especially; since these scales are not painful so spraying the formulation would leave a thin layer of a residue on the skin, liquid or powder, making the invisible delivery system more patient-friendly. More clinical studies on human psoriatic skin need to be conducted as well as most studies use only animal models or cell lines to mimic psoriatic skin. While very few articles conducted their experiments on real psoriasis’ patients

Conclusively, better skin penetration, deposition, and lower systemic absorption are the targets to completely control psoriatic plaques. Using suitable nanocarriers along with the optimum active ingredient may achieve the goal and make this disease a minor controllable condition that neither affects patients physiologically nor psychologically.
